# COVID-19 case counts and COVID-19 related Emergency Department visits: differences by immigration status, March-December 2020

**DOI:** 10.1186/s12889-022-14345-9

**Published:** 2022-10-26

**Authors:** Annie Ro, Michael Pham Huynh, Tim A. Bruckner, Senxi Du, Andrew Young

**Affiliations:** 1 UC Irvine Program in Public Health, Department of Health, Society, and Behavior Anteater Instruction and Research Building (AIRB), Room 2036 653 E. Peltason Road, 92597 Irvine, CA USA; 2 UC Irvine Program in Public Health, Department of Health, Society, and Behavior Center for Population, Inequality, and Policy, Anteater Instruction and Research Building (AIRB), 653 E. Peltason Road, 92597 Irvine, CA USA; 3 Keck School of Medicine, University of Southern California, Los Angeles, CA USA; 4 Division of Geriatric, Hospital, Palliative and General Internal Medicine, Department of Medicine, Keck School of Medicine of USC, Los Angeles, CA USA

**Keywords:** Disparities, Emergency department, Immigrants, COVID-19

## Abstract

**Background:**

Undocumented immigrants face barriers to health care access, which may have been exacerbated during the early days of the COVID-19 pandemic. We test whether undocumented immigrants in Los Angeles County accessed COVID-19 related medical care by examining their Emergency Department (ED) patterns through high and low periods of COVID-19 infection. If undocumented immigrants were underutilizing or foregoing health care, we expect null or weaker associations between COVID-19 cases and COVID-19 related ED visits relative to Medi-Cal patients.

**Methods:**

We analyzed all ED visits to the Los Angeles County + University of Southern California (LAC + USC) Medical Center between March - December 2020 (n = 85,387). We conducted logistic regressions with Los Angeles County weekly COVID-19 case counts as our main independent variable and an interaction between case counts and immigration status, stratified by age (over and under 65 years).

**Results:**

We found that undocumented immigrants under 65 years old had a higher odds for a COVID-19 related ED visit compared to Medi-Cal patients and that both undocumented and Medi-Cal patients had higher odds of a COVID-19 related ED visit as COVID-19 cases in Los Angeles County increased. For patients over 65 years, Medi-Cal patients actually had a weaker association between ED visits and county COVID-19 counts; as COVID-19 case counts rose, the odds of a COVID-19 related ED visit increased for the undocumented patients.

**Conclusion:**

While the overall likelihood of undocumented patients having a COVID-19 related ED visit varies compared to Medi-Cal patients - for younger patients, the odds is higher; for older patients, the odds is lower - it does not appear that undocumented patients underutilized the ED during the early COVID-19 pandemic relative to Medi-Cal patients. The ED may be a viable source of contact for this high-risk population for future outreach.

## Background

The COVID-19 pandemic has laid bare the importance of medical care access, yet access to and utilization of health care varies widely by social position [1]. A number of commentaries have identified undocumented status as a crucial social factor that raises the risk for poor COVID-19 outcomes, due in part to undocumented immigrants’ lack of access to care [2–4]. Undocumented immigrants generally have low levels of insurance coverage and are less likely to have a regular source or care or visit a doctor in the past year [5, 6]. These disparities are tied to their lower socioeconomic resources and concerns interacting with government-related institutions because of their immigration status.

The pandemic may have exacerbated these barriers to care, especially for visits to the Emergency Department (ED) [7]. ED visits drastically fell early in 2020, as stay-at-home orders proliferated and patients avoided seeking health care for non-COVID-19 related conditions. Hospital surveillance data indicated a 42% decline in ED visits nationwide in March and April 2020 compared to the same period in 2019, a mean of 2.1 million fewer visits per week. This drop may have been even steeper for undocumented immigrants, as they were specifically excluded from federal COVID-19 economic relief [8], which could help alleviate costs of heath-care seeking. Latinos, who make up the majority of the undocumented population in the United States, were also the most negatively affected economically by the pandemic [9]. The higher barriers to care are especially concerning because undocumented immigrants are more likely to be infected with COVID-19 and experience poorer outcomes. Multiple studies have found Latinos, who make up the majority of the undocumented population, to have higher odds of being infected or hospitalized due to COVID-19 compared to non-Hispanic Whites, especially in the early days of the pandemic [10–12].

However, establishing whether undocumented immigrants are underutilizing COVID-19 related medical care is challenging, as data on this population are scarce and there is no baseline data to assess COVID-19 related medical need. One way to test if undocumented immigrants are adequately accessing COVID-19 related medical care is to examine how sensitive their utilization patterns are to high and low periods of COVID-19 infection in the community relative to documented patients. We focus on ED visits as our measure of utilization, as undocumented immigrants are less likely to have a regular source of primary care [6]. ED utilization has also been used for other chronic diseases to study the differential burden of disease, as well as the differential impacts of structural factors such as health care access [13].

This paper examines undocumented immigrants’ potential ED underutilization relative to documented patients by examining the relationship between county COVID-19 case counts and COVID-19 related ED visits by immigration status in Los Angeles County between March 2020 and December 2020. This time period includes relatively low case rates through September 2020 as well as the first half of the massive second wave in Southern California that peaked in December 2020. We assume that COVID-19 case counts represent community-level need for COVID-19 related medical care; the higher the case counts, the more COVID-19 related care should be seen in health care settings. If undocumented immigrants were experiencing unique barriers that deter their help-seeking, we expect immigration status to modify the relationship between COVID-19 case counts and COVID-19 cases in the ED. Specifically, undocumented patients will have a weaker relationship between COVID-19 cases and COVID-19 related ED visits relative to documented patients. If undocumented immigrants did not experience unique barriers to health care, periods of high infection should correspond with higher levels of ED utilization for COVID-19 related diagnoses for both undocumented and documented patients.

## Methods

### Data

We analyzed all ED visits to the Los Angeles County + University of Southern California (LAC + USC) Medical Center between March 20th, 2020 and December 31st, 2020 (n = 85,387). The patient records were extracted from LAC + USC Medical Center’s Vizient Health System Data records, a hospital billing and administrative claims database. We limited our sample to visits of patients 18 years of age and older who were either insured by restricted Medi-Cal or full-scope Medi-Cal, leaving us with a sample of 49,574 ED encounters over the 10-month period. All data were de-identified to conform to Health Insurance Portability and Accountability Act (HIPAA) requirements. All project activities were reviewed and approved by the University of Southern California Institutional Review Board (HS-19-00890), which served as a reliance for the University of California, Irvine Institutional Review Board.

### Variables

*COVID-19 ED visit.* Our dependent variable was whether the ED encounter was a visit with a COVID-19 diagnosis, which was defined using the International Classification of Diseases, Tenth Revision (ICD-10) code, U07.1, for any of the first 20 ICD-10 diagnoses of a patient encounter.

*Immigration Status*. We used primary payer status of the encounter to approximate immigration status. We coded a patient as having undocumented status if restricted-scope Medi-Cal was the primary payer source for the ED encounter. In California, restricted-scope Medi-Cal, or “Emergency Medi-Cal,” covers the cost of emergency services for low-income patients who are ineligible for Medi-Cal because of their immigration status (i.e., they are not U.S. nationals, citizens, or lawful permanent residents). Restricted Medi-Cal was also expanded to include COVID-19 testing and treatment at no cost to the patient [14]. We follow the lead of previously published work that has used this insurance coverage type as a proxy for undocumented status [15, 16]. We chose full-scope Medi-Cal (hereafter referred to as Medi-Cal) patients as a comparison group of low-income patients who are either U.S.-born or foreign-born with authorized status. Because of the program’s “lawful status” requirements, we can assume that the Medi-Cal patients are either citizens or lawfully residing in the United States.

*COVID-19 Case Counts*. This data came from the California Department of Public Health through the California Open Data Portal, with no restrictions on public access use. Information in this dataset included COVID-19 cases and deaths by county each day from March 18th, 2020 to December 31st, 2020. We aggregated by week and divided by 1000 to establish weekly LA County case counts per 1000 people. Whereas COVID-19 case counts are not differentiated by immigration status, we would expect the infection rates among undocumented immigrants to mirror those among the county as a whole. Approximately one out of three undocumented immigrants in the state live in “mixed status” households [8], meaning there are few physical or geographic boundaries that would differentiate their risk from the population with lawful status.

*Covariates*. We used the ED visit data to retrieve other demographic variables as covariates, including sex (male (ref.), female), race/ethnicity (non-Hispanic White (ref.), non-Hispanic Black, non-Hispanic Asian, non-Hispanic Other, Hispanic), age, and month of encounter (April (ref.), May, June, July, August, September, October, November, December).

### Analysis

We conducted two logistic regression models to test our hypotheses. In the first, we estimated the log-odds of an ED visit being a COVID-19 visit as a function of prior week COVID-19 cases, immigration status, and covariates (month, race/ethnicity, gender, and age). The second model was identical to the first but included an interaction term for prior week COVID-19 cases and immigration status. We calculated group-specific odds ratios and predicted probabilities, the latter of which we graphed to ease interpretation. We stratified analyses by age (18–64 years of age and 65 year and over) because of the increased severity of COVID-19 among older adults.

## Results

Table 1 provides a descriptive table of all ED visits during the study period. More of the ED visits were from men (53.7%) than women (46.3%). For undocumented patients, those who were between 45 and 54 years old made up the highest percent of all visits (30.3%), while 18–34 years olds made up the highest percent of Medi-Cal patients (33.1%). The vast majority of undocumented patients were Hispanic (94%), compared to over half of Medi-Cal patients (56.4%). Visits with a COVID-19 diagnosis made up a higher proportion of ED visits for undocumented patients (5.5%) than Medi-Cal patients (3.7%). The median weekly COVID-19 case count the period was 9,611 per week (range 842 − 93,747).

**Table 1:** Demographic Characteristics of Emergency Department Visits from March 20, 2020 to December 31, 2020 at LAC + USC Medical Center

	Undocumented (n = 19,615)		Medi-Cal (n = 38,125)		Total (n = 57,740)	
	N	%	N	%	N	%
**Sex**						
Female	10,593	54.0%	16,114	42.3%	26,707	46.3%
Male	9,022	46.0%	22,011	57.7%	31,033	53.7%
**Age**						
18–34	2,169	11.1%	12,607	33.1%	14,776	25.6%
35–44	5,042	25.7%	5,831	15.3%	10,873	18.8%
45–54	5,949	30.3%	6,430	16.9%	12,379	21.4%
55–64	4,051	20.7%	7,354	19.3%	11,405	19.8%
65+	2,404	12.3%	5,903	15.5%	8,307	14.4%
**Race/Ethnicity**						
Non-Hispanic White	84	0.4%	1,892	5.0%	1,976	3.4%
Non-Hispanic Black	80	0.4%	6,866	18.0%	6,946	12.0%
Non-Hispanic Asian	456	2.3%	1,417	3.7%	1,873	3.2%
Non-Hispanic Other	561	2.9%	6,463	17.0%	7,024	12.2%
Hispanic	18,434	94.0%	21,487	56.4%	39,921	69.1%
**COVID-19 Diagnosis**						
Yes	1,071	5.5%	1,416	3.7%	2,487	4.3%
No	18,544	94.5%	36,709	96.3%	55,253	95.7%

Table 2 provides the results of our logistic regression models for patients 18–64 years of age. For every new COVID-19 case per 1000 people in the county, the odds of an ED visit being related to COVID-19 increased by 1% (OR = 1.01, 95% CI = 1.01–1.02) for this age group. Undocumented patients were also more likely to have a COVID-19 related ED visit than Medi-Cal patients during the study period (OR = 1.37, 95% CI = 1.24–1.52). Model 2 includes the interaction term between COVID-19 cases and immigration status. The interaction was not significant, meaning that immigration status did not modify the relationship between COVID-19 cases and ED visits. The odds of a COVID-19 related ED visit increased by 1% for every new COVID-19 case per 1000 people in the county for both undocumented and Medi-Cal patients (Medi-Cal patients: OR = 1.01, 95% CI = 1.01–1.02; undocumented patients: OR = 1.01, 95% CI 1.00-1.02).

**Figure 1:**
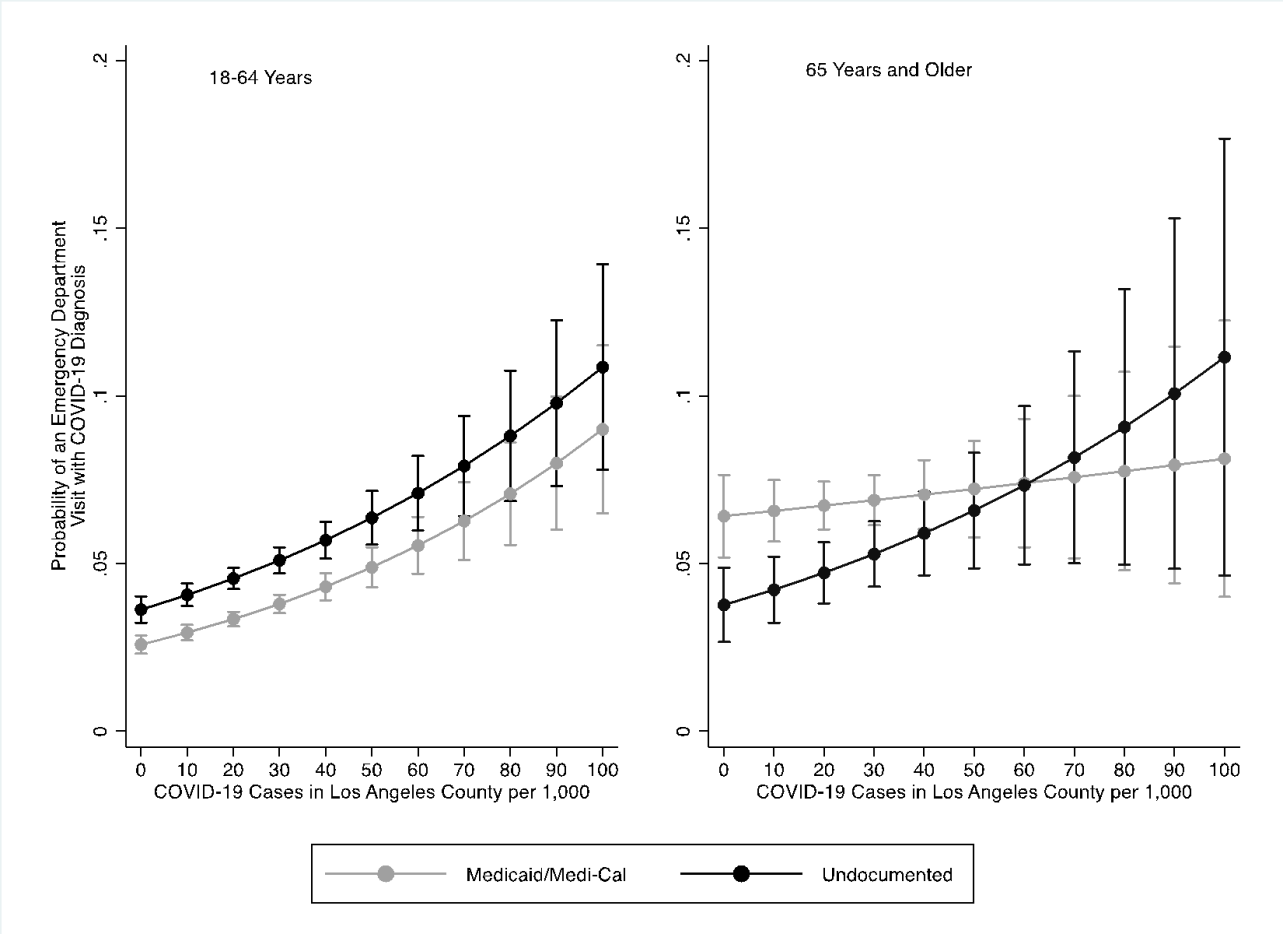
Predicted Probability of COVID-19 related ED visit at LAC + USC Medical Center by immigration status and age, March 2020-December 2020

Figure 1 illustrates the predicted probabilities of a COVID-19 related ED visit by COVID case counts for undocumented immigrant and Medi-Cal patients by age. For patients 18–64 years, undocumented patients had an elevated predicted probability of a COVID-19 related ED visit compared to Medi-Cal patients whether community-level infection was high or low. Yet the probability of a COVID-19 ED visit rises similarly over increasing COVID-19 case counts for both undocumented and Medi-Cal patients.

**Table 2:** Odds of a COVID-19 diagnosed ED visit at LAC + USC Medical Center March 2020-December 2020, patients 18–64 years old

	Model 1					Model 2			
	OR	95% CI		*p*		OR	95% CI		*p*
**Prior week COVID-19 cases (per 1000)**	1.01	1.01	1.02	**		1.01	1.01	1.02	**
**Status**									
Medi-Cal	Ref.					Ref.			
Undocumented	1.37	1.24	1.52	**		1.42	1.25	1.63	**
**Undocumented x Prior week COVID-19 cases (per 1000)**						1.00	1.00	1.00	
**Month**									
March	0.15	0.07	0.33	**		0.15	0.07	0.33	**
April	Ref.					Ref.			
May	1.37	1.08	1.75	*		1.37	1.08	1.75	*
June	1.93	1.54	2.42	**		1.93	1.54	2.42	**
July	1.59	1.26	2.01	**		1.59	1.26	2.01	**
August	0.96	0.75	1.23			0.96	0.75	1.23	
September	0.57	0.43	0.75	**		0.57	0.43	0.75	**
October	0.39	0.29	0.53	**		0.39	0.29	0.53	**
November	0.88	0.68	1.13			0.88	0.68	1.13	
December	1.69	1.23	2.33	**		1.69	1.23	2.33	**
**Race/Ethnicity**									
Non-Hispanic White	Ref.					Ref.			
Non-Hispanic Black	0.86	0.58	1.30			0.86	0.58	1.30	
Non-Hispanic Asian	0.89	0.51	1.54			0.89	0.51	1.55	
Non-Hispanic Other	1.59	1.08	2.33	*		1.59	1.08	2.33	*
Hispanic	2.37	1.66	3.40	**		2.38	1.66	3.40	**
**Sex**									
Male	Ref.					Ref.			
Female	0.80	0.73	0.88	**		0.80	0.73	0.88	**
**Age**									
18–34	Ref.					Ref.			
35–44	1.14	0.99	1.31			1.14	0.99	1.31	
45–54	1.40	1.22	1.60	**		1.40	1.22	1.60	**
55–64	1.41	1.23	1.62	**		1.41	1.23	1.62	**

** p < .01; *p < .05

Table 3 provides the results for older patients. In Model 1, prior week COVID-19 cases were not associated with COVID-19 diagnosed ED visit and undocumented immigrants had significantly lower odds of having a COVID-19 related visit than Medi-Cal patients (OR = 0.56, 95% CI = 0.41–0.77). In Model 2, the interaction between prior week COVID-19 cases and immigration status was significant (OR = 1.01, 95% CI = 1.00-1.02) indicating effect modification. For Medi-Cal patients, where was no significant relationship between COVID-19 cases and COVID-19 diagnosed ED visits (OR = 1.00, 95% CI = 0.99–1.02). For undocumented patients, however, there was a significant relationship (OR = 1.01, 95% CI = 1.00-1.03).

**Table 3:** Odds of a COVID-19 diagnosed ED visit at LAC + USC Medical Center March 2020-December 2020, patients 65 years and older

	Model 1					Model 2			
	OR	95% CI		*p*		OR	95% CI		*p*
**Prior week COVID-19 cases (per 1000)**	1.01	1.00	1.01			1.00	0.99	1.01	
**Status**									
Medi-Cal	1.00					1.00			
Undocumented	0.73	0.58	0.91	*		0.56	0.41	0.77	**
**Undocumented x Prior week COVID-19 cases (per 1000)**						1.01	1.00	1.02	*
**Month**									
March	0.16	0.05	0.53	**		0.16	0.05	0.53	**
April	1.00					1.00			
May	1.15	0.74	1.80			1.15	0.74	1.81	
June	0.94	0.60	1.48			0.96	0.61	1.51	
July	1.03	0.66	1.61			1.04	0.66	1.63	
August	0.65	0.40	1.04			0.65	0.40	1.05	
September	0.51	0.31	0.83	*		0.51	0.31	0.84	*
October	0.46	0.28	0.77	**		0.47	0.28	0.78	**
November	0.48	0.29	0.80	*		0.49	0.29	0.81	*
December	2.55	1.42	4.58	**		2.55	1.42	4.58	**
**Race/Ethnicity**									
Non-Hispanic White	1.00					1.00			
Non-Hispanic Black	0.97	0.41	2.28			0.99	0.42	2.33	
Non-Hispanic Asian	1.15	0.48	2.76			1.16	0.48	2.76	
Non-Hispanic Other	2.95	1.32	6.57	*		2.96	1.33	6.59	*
Hispanic	2.53	1.17	5.47	*		2.55	1.18	5.50	*
**Sex**									
Male	1.00					1.00			
Female	0.92	0.76	1.12			0.91	0.75	1.11	
**Age**									
18–34	1.00					1.00			
35–44	1.44	1.11	1.86	*		1.45	1.12	1.87	**
45–54	1.58	1.27	1.98	*		1.61	1.28	2.01	**

** p < .01; *p < .05

Figure 1 illustrates the interaction. The Medi-Cal patients were less sensitive to rising COVID-19 cases than undocumented immigrant patients. For undocumented patients, the probability of a COVID-19 related ED visit increased as case count increased. Conversely, the predicted probabilities of a COVID-19 related visit remained relatively flat over increasing COVID-19 cases for Medi-Cal patients. Older undocumented immigrants had lower odds of having a COVID-19 diagnosed ED visit until 60 cases per 1,000, where their predicted probabilities surpassed those of Medi-Cal patients. This cross-over point is in the 93rd percentile of COVID-19 cases in the county, however, suggesting that older undocumented immigrants had lower odds of a COVID-19 related ED visit for the majority of the range of COVID-19 cases.

## Discussion

This paper examined the relationship between prior week COVID-19 cases in Los Angeles County and COVID-19 related ED visits in the largest safety-net hospital in the county. We assumed county COVID-19 case counts represented the population-based need for health care services for both undocumented and Medi-Cal patients. Younger undocumented immigrants did not underutilize the ED for COVID-19 related needs relative to Medi-Cal patients; both undocumented and Medi-Cal patients had more COVID-19 ED visits as infection rates rose in the county. We found, however, that younger undocumented immigrants were more likely to have a COVID-19 diagnosed ED visit than Medi-Cal patients. Our finding suggests higher COVID-19 burden among younger undocumented immigrants, but it does not appear that they are avoiding medical care in light of their elevated infections.

While younger undocumented patients had higher COVID-19 related ED visits relative Medi-Cal patients, the opposite was true for older undocumented patients. For the majority of the case count range, undocumented immigrants’ odds of a COVID-19 ED visit were lower than their Medi-Cal counterparts. However, their COVID-19 related ED visits increased as case counts increased, suggesting that when the community needs for COVID-19 medical services were higher, older undocumented immigrants utilized the ED appropriately. Unexpectedly, we found the Medi-Cal patients to be less sensitive to case counts. The reason for this are unclear but one possibility is that Medi-Cal patients in this hospital faced consistently elevated COVID-19 risk because of pre-existing conditions even as the county case counts fluctuated. Further studies should further characterize whether this effect would continue beyond the phase of the pandemic reflected in our data.

To our knowledge, our paper is the first to test the relationship between COVID-19 medical need and COVID-19 related ED visits for undocumented immigrants. Our results differ from other papers that suggest undocumented immigrants’ ED use would have underutilized the ED relative to Medi-Cal patients. Devillanova et al. found undocumented immigrants in Italy to have a reduction in visits for severe respiratory illness after the lockdown in that country [17], implying that undocumented immigrants were not seeking medical care for symptoms indicative of COVID-19 during the lock-down. Ro et al. found that undocumented immigrants’ overall ED use dropped lower to lower-than-expected levels during the first stay-at-home orders in the Spring 2020, even more so than for Medi-Cal patients [18]. Our results differ from these findings in that we specifically examine COVID-19 diagnosed ED visits. Whereas ED use for other conditions may have decreased, help seeking in the ED for COVID-19 medical needs appears to be unique from ED visits generally.

## Conclusion

Overall, we found that undocumented immigrants did not seem to underutilize the ED to address their COVID-19 related medical care needs relative to Medi-Cal patients. While their overall levels of COVID-19 related ED visits varied compared to Medi-Cal patients – higher for younger undocumented patients, lower for older undocumented patients – their utilization increased as county case counts increased. While many researchers are concerned that undocumented immigrants may be marginalized in COVID-19 related outreach or intervention [19], our results suggest that the ED can be a viable source of contact with this high-risk population. We note some caveats of our findings, however. First, we only had COVID-19 case counts for the entire population and not separated by ethnicity or immigration. While the level of COVID-19 infection among undocumented immigrants is unknown, it is likely comparable to the general county population. Our findings may also be specific to the geographic location. Los Angeles County has been especially active in expanding access to care to undocumented immigrants. For example, My Health LA is a county program that provides primary and specialty care coverage to low-income immigrant, regardless of citizenship status. As a result, undocumented immigrants in Los Angeles may already be well integrated in the local health system and have had fewer barriers to accessing care during the pandemic than other undocumented populations without the same level of access. Future work could replicate this analysis in other areas without comparable programs in place.

## Data Availability

The datasets generated and/or analyzed during the current study are not publicly available because they contain PHI but are available from the corresponding author on reasonable request.

## List of abbreviations

COVID-19: Coronavirus disease 2019
ED: Emegency Department
LAC + USC: Los Angeles County + University of Southern California Medical Center
ICD-10: International Classification of Diseases, Tenth Revision
